# The Effect of Haematocrit on Measurement of the Mid-Infrared Refractive Index of Plasma in Whole Blood

**DOI:** 10.3390/bios11110417

**Published:** 2021-10-25

**Authors:** David J. Rowe, Daniel R. Owens, Suzanne L. Parker, Saul N. Faust, James S. Wilkinson, Goran Z. Mashanovich

**Affiliations:** 1 Zepler Institute for Photonics and Nanoelectronics, University of Southampton, Southampton SO17 1BJ, UK; jsw@orc.soton.ac.uk (J.S.W.); g.mashanovich@soton.ac.uk (G.Z.M.); 2 Faculty of Medicine and Institute for Life Sciences, University of Southampton, Southampton SO16 6YD, UK; d.owens@soton.ac.uk (D.R.O.); s.faust@soton.ac.uk (S.N.F.); 3 NIHR Southampton Clinical Research Facility and Biomedical Research Centre, University Hospital Southampton NHS Foundation Trust, Southampton SO16 6YD, UK; 4 UQ Centre for Clinical Research, The University of Queensland, Herston, QLD 4029, Australia; suzanne.parker@uq.edu.au

**Keywords:** mid-infrared spectroscopy, biofluid analysis, bioanalytical validation, point-of-care sensing

## Abstract

Recent advances suggest that miniaturised mid-infrared (MIR) devices could replace more time-consuming, laboratory-based techniques for clinical diagnostics. This work uses Fourier transform infrared spectroscopy to show that the MIR complex refractive index of whole blood varies across a range of haematocrit. This indicates that the use of an evanescent measurement is not sufficient to optically exclude the cellular content of blood in the MIR, as previously assumed. Here, spectral refractive index data is presented in two ways. First, it is given as whole blood with varying haematocrit. Second, it is given as the percentage error that haematocrit introduces to plasma. The maximum error in the effective plasma refractive index due to the haematocrit of healthy adults was 0.25% for the real part n and 11% for the imaginary part k. This implies that calibration measurements of haematocrit can be used to account for errors introduced by the cellular content, enabling plasma spectra and analyte concentrations to be indirectly calculated from a whole blood sample. This methodological advance is of clinical importance as plasma concentration of analytes such as drugs can be determined using MIR without the preprocessing of whole blood.

## 1. Introduction

Mid-infrared absorption spectroscopy is a well-established measurement technique that characterises chemical species through the identification of individual molecular bonds, which have characteristic vibrational and rotational resonances in the spectral region of wavelengths from 2 µm to 15 µm (wavenumbers 650–5000 cm^−1^). From the Beer-Lambert Law, absorption strength is proportional to the concentration of each vibrating bond. The unique combinations of absorption peaks and their relative magnitudes for each molecule can be thought of as a spectral fingerprint. 

There are many different configurations that can be used to implement MIR spectroscopy. The O-H bond of water is highly absorbing in this spectral region, therefore aqueous sensing measurements tend to use evanescent methods, where a sample is placed upon a crystal to interact with an incident beam totally and internally reflected within the crystal, rather than transmission methods where the incident beam passes through the entire sample. This reduces the effective interaction length from millimetres to microns so that low-concentration solutes can be detected in the presence of the highly absorbing aqueous background.

Techniques for performing evanescent MIR measurements include attenuated total reflection-Fourier transform infrared (ATR-FTIR) [1,2,3,4] and waveguide spectroscopy [5,6,7,8]. These methods have been applied to a broad variety of sensing applications. Gas sensing applications include aerosol [9], methane [10], and carbon dioxide [11] detection. Biological applications include protein characterisation [12,13] and biofilm analysis [14]. Chemical-sensing applications include hydrocarbon detection in water [15], differentiating between salt solutions [16], and the compositional analysis of organic solvent mixtures [17,18]. An exemplar application for medical sensing would be the rapid detection of analgesics directly from biological tissues such as blood, as discussed in detail in a previous article on the MIR refractive index of blood [19].

A measurement technique that can exclude or account for the cellular content of blood is desirable for rapid point-of-care diagnostics because it simplifies the measurement procedure. The majority of clinical blood analyses require blood plasma or blood serum, which are obtained by centrifuging whole blood. These processes require laboratory facilities and trained personnel and are not simple to miniaturise. Conversely, methods that can directly analyse whole blood, such as a blood glucose fingerprick measurement, can be performed by non-specialist users at the point of care. MIR specificity derives from the optical properties of the sample, meaning that, if the sample can be delivered to the sensor, which is feasible for the non-expert user, then the measurement procedure can be automated. 

MIR measurements can combine the advantage of simplicity inherent to whole blood measurements with the ability to extract clinically useful information from plasma by optically excluding the cellular content of blood. For example, plasma is commonly used for drug testing. In cases of analgesic poisoning, the plasma concentration of the drug is a determining factor in clinical decision making [20,21]. 

MIR technology compares favourably with current state-of-the-art laboratory methods for drug detection due to its potential for miniaturisation for point-of-care sensing. Such methods include immunoassays [22], liquid chromatography–ultraviolet spectroscopy (LC-UV) [23] and liquid chromatography–mass spectrometry (LC-MS) [24,25]. Each of these methods require significant sample preparation by trained laboratory personnel. One aspect of sample preparation is to remove cellular content to negate the impact of haematocrit. This paper shows how to numerically remove the impact of haematocrit from MIR measurements without physically removing cells from the sample.

From the first publications on integrated optics sensing, it has been assumed that evanescent absorption measurements of liquid suspensions are unaffected by suspended bodies such as cells because they do not interact significantly with the evanescent field [26,27], offering the potential to measure blood plasma without removing the red blood cells. This field decays exponentially into the analyte away from the ATR element surface on the scale of spectral wavelength; for example, by several microns in the mid-infrared, whereas cells are at least an order of magnitude larger. By varying the haematocrit of whole blood from a single donor, it is possible to empirically test this hypothesis. In contrast to MIR measurements, Mitchell used a shorter wavelength (λ ≈ 500 nm) [26]. This proportionally reduced the penetration depth and, therefore, the interaction with, the cellular content of whole blood. The functional and fingerprint regions of the mid-infrared spectrum can be used to obtain more specific molecular information for the applications listed above, though are more likely to be affected by haematocrit because of the increased penetration depth caused by the longer wavelength.

This article provides the complex refractive index spectra for whole blood at a range of haematocrit levels. It also presents an error spectrum, where the change in refractive index due to haematocrit is calculated as a percentage error compared to plasma. This enables the identification of the regions where haematocrit could interfere with spectral interpretation. Additionally, it is anticipated that this information will be useful for photonics engineers designing and fabricating MIR devices for clinical applications, especially as these progress from research laboratory methods to higher technology readiness levels. 

Bioanalytical validation is the complete process for demonstrating that a technique can perform pharmacokinetic, toxicokinetic, or biomarker concentration evaluation. The US Food and Drug Administration (FDA) and European Medicines Agency (EMA) provide guidance [28,29] for this process, however both are broadly similar, therefore the present discussion is restricted to the FDA guidelines. Haematocrit is considered in the context of the matrix effect: a technique is required to either demonstrate that it is invariant with changing haematocrit, or else it can account for differences between high and low haematocrit. In practice, this is demonstrated by performing calibrated quality control (QC) measurements at low and high extremes of haematocrit. For the analyte under test, which, in the context of MIR measurements, could be the strength of a group (“fingerprint”) of absorption peaks, QCs are mandated by the FDA at three concentrations: three times the lower limit of quantification, 0.8 times the upper limit of quantification, and one medium value in between the two.

Haematocrit levels vary in healthy and diseased states, meaning that any measurement technique must account for this. In health, haematocrit is usually 35–48%, with males having slightly higher values than females. Critically ill adult patients can record low haematocrit (20–30%) [30], which can be due to a disease state or medical interventions, such as fluid resuscitation. Haematocrit can rise to approximately 60% in individuals living at an altitude in response to relative hypoxia [31]. Haematocrit also varies with age, as neonatal infants have higher values (44–64%), before dropping slightly in early childhood and then rising to reach normal adult values after puberty [32]. Haematocrit can also rise or fall in different disease states. Raised haematocrit, known as polycythaemia, may be seen in neonatal infants in response to intra-uterine hypoxia, and the resultant raised blood viscosity can lead to organ hypoperfusion with lasting consequences if not treated. Raised haematocrit is also seen in older children and adults who have diseases causing tissue hypoxia and also in some bone marrow diseases. Low haematocrit may be due to an inadequate production of red cells, an increased loss of red cells, or blood dilution. 

A method for calculating complex refractive index spectra from non-polarised ATR-FTIR spectroscopy was reported previously [19]. This method was used to calculate the MIR refractive index spectra of whole blood and analgesics, where it was suggested that the cellular content of blood did not interact with the evanescent sensing field, therefore the measured refractive index would effectively be that of plasma. The method for calculating refractive index was theoretically and experimentally simpler than polarised measurement regimes, and was implemented using an empirical calculation of penetration depth. This requires a calibration measurement of a substance with a known refractive index over a wide wavelength range. For whole blood measurements, water was used for the calibration because it has been comprehensively characterised [33] and is the single largest component of blood. Reference to these complex refractive index spectra has been made in articles on MIR device design [34,35,36] and in studies of the properties of biological tissues [37,38]; the effect of varying haematocrit on plasma spectra reported here is expected to allow for an improved interpretation.

## 2. Materials and Methods

Whole blood was collected according to standard clinical research practice, including the current revision of the Declaration of Helsinki 2008 and in full conformity to the guidelines for Good Clinical Practice. Informed consent was obtained from the participant. Samples were collected according to NHS research ethics approval managed by the Southampton Research Biorepository (REC reference 17/NW/0632). The samples were labelled anonymously and were not stored after characterisation. 

### 2.1. Chemicals and Reagents 

20 mL of whole blood was collected with lithium heparin vacutainer tubes (Becton, Dickinson and Company, USA) by venepuncture of a single healthy volunteer. The samples were used within two hours from collection. Isopropanol (99.9% purity, Merck, UK) and deionised (DI) water (Pico 10T2, Triple Red, UK) were used to clean the FTIR instrument.

### 2.2. Modification of Haematocrit

Haematocrit was measured using a XN9000 automated analyser (Sysmex Europe GmbH, Germany) for complete blood count. Whole blood was prepared at a range of haematocrit levels both above (50%, 60%, and 70%) and below (20%, 30%, and 40%) the donor’s level. 

#### 2.2.1. Protocol for Reducing Haematocrit

1. Measure initial haematocrit (HCT);
2. Calculate additional volume of plasma to reduce HCT to desired level using Equation (1):
  (1) $$\Delta v_{plasma}=\left(\frac{H_{initial}}{H_{target}}-1\right)v_{sample},$$
  where $\Delta v_{plasma}$ is the required change in the volume of plasma, $H_{initial}$ and $H_{target}$ are the initial and target haematocrits and $v_{sample}$ is the volume of the whole blood sample to be modified. Positive $\Delta v_{plasma}$ requires plasma to be added; negative $\Delta v_{plasma}$ means plasma should be removed;

3. Separate plasma from whole blood stock (Eppendorf centrifuge: 3000 rpm, 10 min).
4. Add plasma to whole blood and gently mix with rotation mixer for 30 min.

#### 2.2.2. Protocol for increasing haematocrit

1. Measure initial HCT;
2. Calculate the reduction in volume of plasma required for the desired HCT level using Equation (2);
3. Gently centrifuge whole blood (1000 rpm, 15 min) to separate plasma and red blood cells;
4. Pipette off the difference in plasma;
5. Gently mix with rotation mixer for 30 min.

### 2.3. ATR-FTIR Measurements

Mid-infrared measurements were taken with an Alpha II FTIR spectrometer with ATR attachment (both Bruker, Germany). The ATR element was a single reflection diamond crystal. Spectra were taken at 4 cm^−1^ resolution in the wavenumber range 4000–600 cm^−1^ using 32 averaged repeats per measurement. A pipette was used to deliver 30 μL of each sample onto the crystal surface. Each sample was characterised in triplicate and the mean was used for subsequent calculations. The spectra were baseline-corrected and had the CO_2_ absorption peaks removed. 

## 3. Results

**Figure 1:**
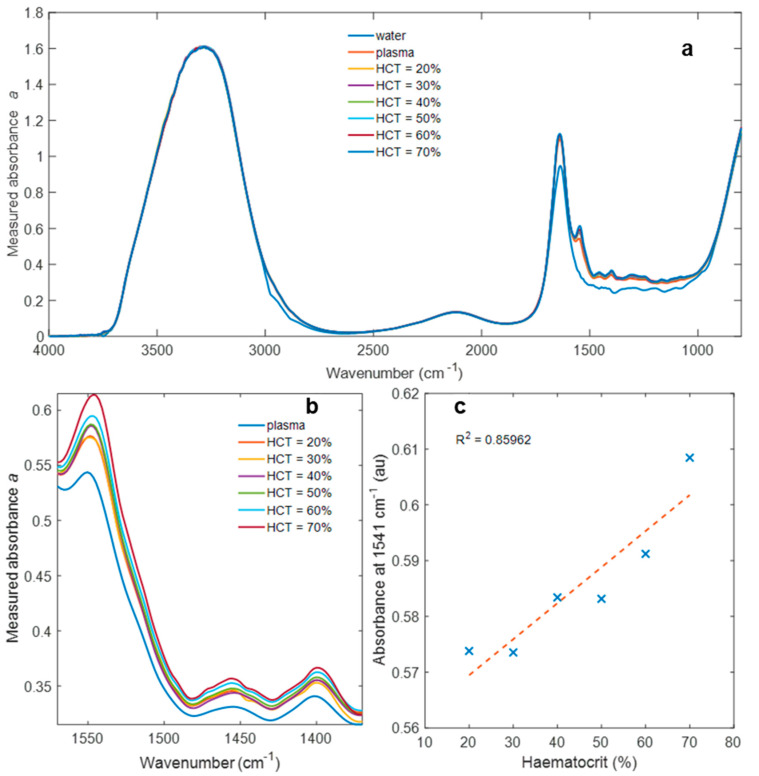
Absorbance spectra of (**a**) DI water, plasma and whole blood with haematocrit in the range 20–70%, (**b**) plasma and whole blood with haematocrit in the range 20–70% over a more limited frequency range (1370–1570 cm^−1^), and (**c**) absorbance at 1541 cm^−1^ with respect to haematocrit.

**Figure 2:**
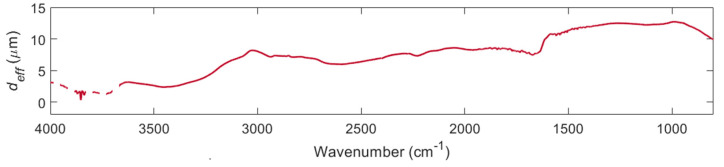
Empirical effective penetration depth *d_eff_* calculated from the measured absorbance and literature *k* values of water: The dashed trace at wavenumbers > 3700 cm^−1^ show the region where *d_eff_* has been calculated from a ratio where both quantities are approximately equal to zero and therefore cannot be relied upon.

Figure 1 shows absorbance spectra in the range of 800–4000 cm^−1^ for DI water and whole blood with haematocrit levels in the range of 20–70%. The spectra have been normalised at the O-H stretch absorption peak at 3300 cm^−1^. There are differences between the spectra at the second highest absorption peak of water, with the O-H bending absorption close to 1600 cm^−1^. These are not removed by normalisation because of band distortion, which is commonly encountered for strongly absorbing samples when using the ATR form of FTIR. Band distortion occurs due to the difference in the refractive index between the two peaks causing a different penetration depth, shown to be a factor of 3 in Figure 2. However, this does not alter the efficacy of the regression procedure used to examine the spectral contribution of haematocrit.

Figure 1b shows the fingerprint region between 1370 and 1570 cm^−1^, where changing haematocrit shows the strongest variations in the measured spectra. Preliminary work examined the difference in absorbance between whole blood and centrifuged plasma using blood from different volunteers. The most significant differences due to the presence or absence of cells were consistently observed in the 1370–1570 cm^−1^ range. Donated blood from a single volunteer has been used throughout this work to minimise the effect of variation in the background plasma measurement used to calculate spectral differences due to the change in haematocrit. 

Figure 1c shows how absorbance varies with respect to haematocrit at the single frequency of 1541 cm^−1^. This absorption peak represents the amide II band, corresponding to N-H bending and C-H stretching. This suggests that ATR-FTIR spectroscopy detects proteins on cell surfaces, which are likely to be the chemical species in closest proximity to the evanescent measurement field as opposed to intracellular species.

Harrick [1] defined the wavelength-dependent relationship between the imaginary part of refractive index *k* and absorption *a* as

(2) $$k\left(\lambda \right)=\frac{\lambda a}{4\pi d\log_{10}e},$$

with wavelength *λ*, path length *d*, and absorbance *a* = −log_10_(*I/I_0_)*, where *I* and *I_0_* are the sample and reference intensities, respectively. Absorbance *a* can be related to concentration and molar absorptivity using the Beer–Lambert law. In conventional spectroscopy, the path length is easily defined, for example, by the dimensions of a cuvette. This is not possible in evanescent techniques such as ATR-FTIR spectroscopy, where the incident beam does not propagate through the sample. However, an effective penetration depth *d_eff_* can be defined for use in Equation (1) as the equivalent thickness of a material that would give the same absorption in transmission. It is possible to derive an empirical effective penetration depth by measuring a sample of known refractive index and rearranging Equation (1) for d, as discussed in detail previously [19]. 

Figure 2 shows the empirical effective penetration depth *d_eff_* calculated from the measured absorbance and literature k values [33] of water by using Equation (2) rearranged for d. The dashed trace at wavenumbers > 3700 cm^−1^ show the region where *d_eff_* has been calculated from a ratio where both quantities are approximately equal to zero, and therefore cannot be relied upon. The effective penetration depth is required for converting ATR-FTIR absorbance values into the complex refractive index.

**Figure 3:**
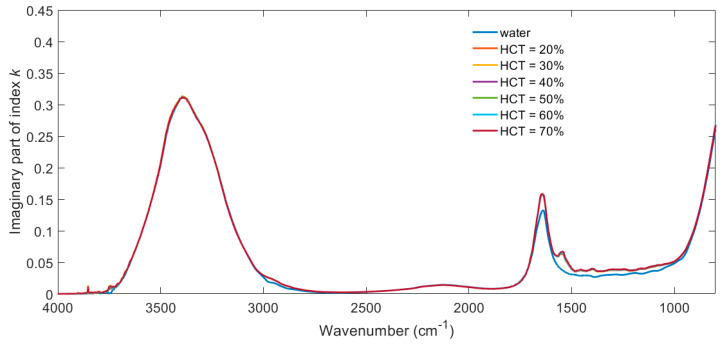
Imaginary part of refractive index spectra k(*λ*) for water and whole blood with haematocrit in the range of 20–70%.

Figure 3 shows the imaginary part of the refractive index k calculated from the absorption data shown in Figure 1. In this case, the path length is taken as the empirical effective penetration depth *d_eff_* shown in Figure 2.

**Figure 4:**
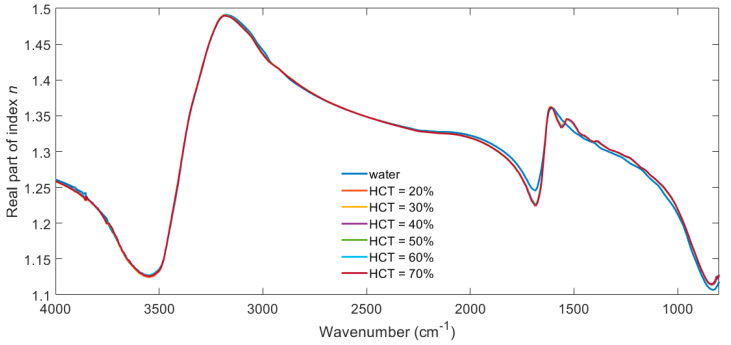
Real part of refractive index spectra n(*λ*) for water and whole blood with haematocrit in the range of 20–70%.

Figure 4 shows the corresponding real part of the complex refractive index n for water and whole blood with haematocrit in the range of 20–70%. These values were calculated from the imaginary part using the Kramers–Kronig Relations, which allow either of the components of a complex function to be derived from the other. The results are obtained in terms of susceptibility and therefore require an offset to obtain refractive index *n*. This offset is taken from a region of low loss refractive index where there are no depolarisation contributions. Here, the offset is taken as the refractive index of water at 2 µm wavelength where whole blood does not contribute any additional spectral features. 

**Figure 5:**
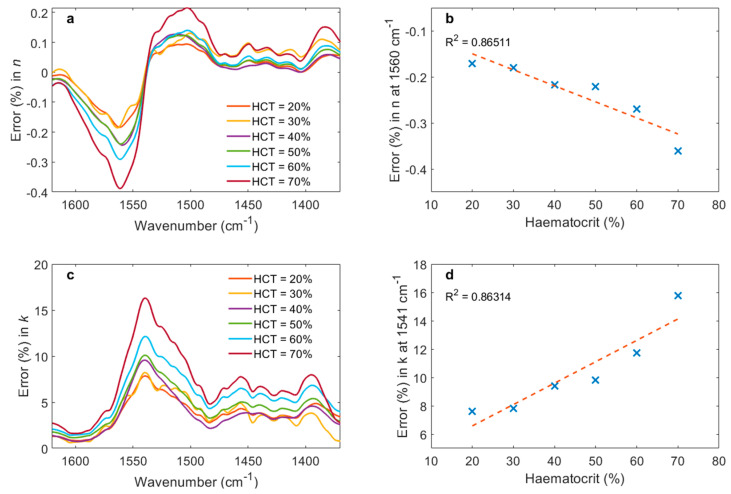
Error in (**a**) real and (**c**) imaginary parts of plasma refractive index due to haematocrit in the range 20–70%: (**b**) shows the maximum error in n, which occurs at 1560 cm^−1^, with respect to haematocrit; (**d**) shows the corresponding behaviour for *k*, which occurs at 1541 cm^−1^.

Figure 3 and Figure 4 are included to illustrate the fact that the complex refractive index of blood is dominated by the contribution of water over the majority of the spectrum. The tabulated form of this data is available at https://doi.org/10.5258/SOTON/D1621 (accessed on 21 October 2021), for example, for use in optical simulations. The spectral contributions due to haematocrit are best demonstrated by calculating the difference between plasma and the different haematocrit samples and plotting the resulting error function over a smaller spectral range, as in Figure 5. 

Figure 5 shows the percentage error of (a) *n* and (b) *k* introduced by whole blood with haematocrit in the of range 20–70% relative to those of plasma, which has had all cellular content removed by centrifugation. The plots show the region of the highest error, which is in the range of 1370–1620 cm^−1^. Figure 5c shows regression analysis for the error of *n* at 1560 cm^−1^, which is the frequency of the highest error. Figure 5d shows the equivalent data for *k*, where the maximum error occurs at 1541 cm^−1^. The data in Figure 5c and d can be used to extrapolate the error in the plasma refractive index due to haematocrit in the healthy adult range of haematocrit = 35–48%. For *n*, this corresponds to error = 0.20–0.25%. For *k*, this corresponds to error = 8.8–11%. The fact that the peaks in error for *n* and *k* occur at different frequencies is an artefact of the relationship between the real and imaginary components of a complex value, where a peak in one corresponds to a zero in the other.

## 4. Discussion

Haematocrit influences evanescent mid-infrared characterisation of plasma in measurements of whole blood, as illustrated in Figure 1. The fact that absorbance varies with haematocrit indicates that cells do at least partially occupy the evanescent field and therefore cannot be assumed to not interfere with such measurements. The main contributions of changing haematocrit are in the fingerprint region between 850 and 1750 cm^−1^, which will interfere with absorption peaks corresponding to proteins, DNA, and drugs.

This information is important for the quantitative processing of spectroscopic data. For example, it is necessary to account for haematocrit in bioanalytical MIR measurements when identifying the spectroscopic signature of a biomarker or when analysing the time-resolved pharmacokinetic response of a patient to a medicine.

This could affect different methods in different ways. A regression algorithm based upon the change in the magnitude of absorption for a drug-specific fingerprint would be required to demonstrate that the change is not due to patient variability in haematocrit. If an exploratory analysis technique, such as principal component analysis, is used to find the spectral regions which dominate the analyte response, the components must be shown to be independent of haematocrit.

The information in Figure 5 demonstrates an alternative: to measure haematocrit independently and correct the refractive index data by subtracting the haematocrit contribution. For MIR bioanalytical validation, this work demonstrates that haematocrit can be validated by manually adding or removing cells from a single sample, rather than recruiting patients with a variety of haematocrit levels and characterising them individually.

The percentage error in *k* is bigger than that of *n* because the absolute values of *k* are significantly lower than those of *n*; cf. Figure 3 and Figure 4 at around 1500 cm^−1^. The absolute values of error are comparable.

Inflammatory and infectious conditions, which increase the number of circulating white blood cells, are unlikely to significantly affect the refractive index spectrum. This is because the number of red blood cells exceeds that of all other cell types by orders of magnitude, thereofre changes in haematocrit dominate any other changes in cell count in the MIR response. 

The utility of the presented method is in removing the effect of haematocrit from MIR measurements. It should be emphasised that evanescent MIR measurements are not well suited for the characterising of cells in suspension, which is the reason for using evanescent measurements for this study. The measurement is restricted to within a few microns of the sensing surface (cf. Figure 2), which is why cellular contributions are mostly but not entirely excluded from the refractive index spectrum. For these reasons, this method is not suitable for detailed studies of red blood cells. Transmission microscopy would be better suited to this task, which is why optical and impedance cytometry form the basis of modern haematology analysers.

There is a measurable difference between the haematocrit of premenopausal women and age-matched men, which has been hypothesised to be because of regular physiologic bleeding, resulting in lower haematocrit and lower viscosity in female blood [39]. For MIR measurements, this effect is likely to dominate any other sex differences because it is manifested in such a large change in composition by volume fraction. Other differences, such as hormone composition, are orders of magnitude smaller. This highlights the need for the careful study design and statistical analysis of MIR data, including consideration of sex and age.

## 5. Conclusions

ATR-FTIR measurements have been used to demonstrate that varying haematocrit has a measurable impact upon the evanescent mid-infrared spectroscopy of blood plasma. The refractive index contribution of the cellular content of blood has been quantified as a percentage error relative to the refractive index of plasma, giving a maximum error of 11% in the imaginary part of index *k* at 1541 cm^−1^. This shows that it is possible to interrogate the optical properties of plasma from a whole blood sample without the need to separate the cellular components from the sample by using an evanescent MIR characterisation technique. This enables plasma-specific applications such as drug quantification with minimal sample preparation. The impact of haematocrit upon regulatory approval for MIR diagnostic devices was discussed, including the need to account for inter-patient variability by sex and age. The complex refractive index spectra have been calculated for blood samples prepared at a range of different haematocrit levels for use in electromagnetic simulations and device optimisations. All data presented in this article are openly available at https://doi.org/10.5258/SOTON/D1621, accessed on 21 October 2021.

## Data Availability

The datasets generated and analysed during the current study are available from the University of Southampton ePrints repository at https://doi.org/10.5258/SOTON/D1621, accessed on 21 October 2021.
